# PD197 Improving The Impact Of Health Technology Assessment Reports: Experience Of The Synthesis In Catalonia

**DOI:** 10.1017/S0266462324004197

**Published:** 2025-01-07

**Authors:** Berta Mestre Lleixà, Rosa Maria Vivanco-Hidalgo

## Abstract

**Introduction:**

To enhance the impact of health technology assessment (HTA) on Catalonia’s health system, a summary product called “synthesis” was developed. It presents the key information from HTA reports. After initial development and pilot testing, we developed a final version. Our aim was to evaluate its impact on decision-making at different levels.

**Methods:**

We prepared an eight-question survey to assess the impact of the 15 syntheses that were published on the Agency for Health Quality and Assessment of Catalonia (AQuAS) website in 2023. We asked about the role of the healthcare professionals answering the questions, why they used the information of the syntheses, and whether they read the associated HTA report later and recommend it. We invited the professionals of the Catalan health system, providers, and payers to answer the survey by email. The survey also was published on the AQuAS website.

**Results:**

We obtained 31 responses. The roles of the professionals who answered the questions were mainly directors and managers of the institutions of the Catalan health system (45.2%), healthcare professionals (32.3%), and care managers (12.9%). Most of them consulted the synthesis to update their knowledge about a specific topic (71.0%), obtain reliable data (35.5%), or support a strategic or professional decision (29.0%). The HTA report related to each synthesis was only consulted in some cases (38.7%) but was recommended in almost all cases (93.5%).

**Conclusions:**

Summarizing the most important information of an HTA report in a new product can be a useful tool for improving the impact of the HTA on the Catalan health system. Professionals who answered the survey agreed that the syntheses helped them in making decisions related to the health technologies.

